# Envirotyping within a multi-environment trial allowed identifying genetic determinants of winter oilseed rape yield stability

**DOI:** 10.1007/s00122-024-04664-3

**Published:** 2024-06-19

**Authors:** Erwan Corlouer, Christopher Sauvage, Magalie Leveugle, Nathalie Nesi, Anne Laperche

**Affiliations:** 1 IGEPP, INRAE, Institut Agro, Université de Rennes, 35650 Le Rheu, France; 2 Syngenta SA France, 1228 Chemin de L’Hobit, 31790 Saint-Sauveur, France; 3 Limagrain, 28 Route d’Ennezat, 63720 Chappes, France

## Abstract

**Key message:**

A comprehensive environmental characterization allowed identifying stable and interactive QTL for seed yield: QA09 and QC09a were detected across environments; whereas QA07a was specifically detected on the most stressed environments.

**Abstract:**

A main challenge for rapeseed consists in maintaining seed yield while adapting to climate changes and contributing to environmental-friendly cropping systems. Breeding for cultivar adaptation is one of the keys to meet this challenge. Therefore, we propose to identify the genetic determinant of seed yield stability for winter oilseed rape using GWAS coupled with a multi-environmental trial and to interpret them in the light of environmental characteristics. Due to a comprehensive characterization of a multi-environmental trial using 79 indicators, four contrasting envirotypes were defined and used to identify interactive and stable seed yield QTL. A total of four QTLs were detected, among which, QA09 and QC09a, were stable (detected at the multi-environmental trial scale or for different envirotypes and environments); and one, QA07a, was specifically detected into the most stressed envirotype. The analysis of the molecular diversity at QA07a showed a lack of genetic diversity within modern lines compared to older cultivars bred before the selection for low glucosinolate content. The results were discussed in comparison with other studies and methods as well as in the context of breeding programs.

**Supplementary Information:**

The online version contains supplementary material available at 10.1007/s00122-024-04664-3.

## Introduction

The main challenge for agriculture consists in ensuring food security while adapting to climate changes and contributing to eco-friendly farming systems. Both will be met through cultural practices adaptations and the development of new crop varieties (Lobell et al. 2008). Indeed, crops must face the effects of climate change characterized by an increase in temperature and CO_2_ concentration but also by the increase of intense climatic phenomena (e.g., droughts, floods or frosts; Bell et al. 2004). In addition, agroecological practices have emerged as a way to produce more food in a sustainable manner by enhancing ecology-based practices (Wezel et al. 2014) and reducing the use of pesticide and chemical inputs. These practices include the use of natural biological control of pests, cover crops, intercropping or cultivar mixtures, in addition to new soil management practices (reduced tillage, …) that increases the complexity of plant-environment interactions (Lamichhane et al. 2018).

In the face of these profound changes, crops need to perform under fluctuating agricultural and climatic conditions. The question of breeding new varieties adapted to a wide range of pedoclimatic and environmental conditions, as well as varieties specifically dedicated to targeted environmental conditions, has arisen. Broadly-adapted varieties correspond to those with stable yields under all environmental conditions, while specifically-adapted varieties can present contrasting yields, depending on the characteristics of the environment.

The wide *versus* specific adaptation can be represented by the seed yield variation observed for a dedicated genotype under a variety of environmental conditions (El-Soda et al. 2014). Yield variation differences exist among genotypes indicating the possibility to breed for stability; however, this remains complex. Indeed, the observed phenotype can be expressed as the sum of a genetic effect (G), an environmental effect (E) defined here as the combination of pedoclimatic conditions and cultural practices, and the *G* × *E* interaction, corresponding to the modification of the phenotypic response to environment between genotypes (Becker and Leon 1988; Malosetti et al. 2016; van Eeuwijk et al. 2016). Breeding programs must therefore consider SY stability to breed for new varieties better adapted to fluctuating environments (Cooper et al. 2020; Snowdon et al. 2021). Classically, breeding programs rely on average performance of genotypes grown under multi-environmental and multi-annual field trials. Although this allows passive selection for a small adaptive effect (Snowdon et al. 2021), this methodology does not allow a direct access to the genetic determinism of seed yield stability (Garin et al. 2020).

A first approach to unravel the genetic determinism of SY stability consists in a comparison of QTL detected using a population trialed across a multi-environmental network. This method is efficient to access to the QTL stability or specificity, but the results remain difficult to interpret (Garin et al. 2020). To gain power in detecting QTL associated to plant adaptation, stability indicators have been developed and used in genetic analyses of stability. For instance, ecovalence indices (Wricke 1962; dos Santos Silva et al. 2021) or AMMI stability values are used to describe genotypic contribution to *G* × *E* and to characterize the adaptability versus stability in different environments (Purchase et al. 2000; Bouchet et al. 2016; Lozada and Carter, 2020; Hassani et al. 2023). Genotypic reaction norms to environmental gradient are also commonly used; they correspond for instance to Finlay and Wilkinson's regression slope (1963) (see Diouf et al. 2020; Xavier et al. 2018; Mangin et al. 2017 for application examples). More recently, linear mixed models and factor analytic models have been developed to consider simultaneously all environments and genotypes in a multi-environment trial (MET) (van Eeuwijk et al. 2010, 2016; Smith et al 2021) and have opened new avenues to detect *G* × *E* interactions and access its genetic determinism (Malosetti et al. 2008; Happ et al. 2021; Chidzanga et al 2022; Chaves et al. 2023). However, without a comprehensive environmental characterization of the MET, the QTL involved in stability is difficult to interpret in terms of the underlying mechanisms of plant adaptation.

Rapeseed is a major worldwide crop cultivated mainly for its seeds oil and meal production, presenting an estimated annual production of 71.3 Mt in 2021 (FAOSTAT 2023). Farmers have more and more difficulties to maintain rapeseed seed yield under adverse environmental conditions (*e.g.*, insect damages during fall, nutritional constraints and abiotic stresses). This leads to a reduction of the cultivated surfaces (− 21% between 2016 and 2022, AGRESTE 2023). Thus, improving seed yield stability is of major concern for oilseed rape (Zandberg et al 2022; Cowling et al 2023). Seed yield (SY) is a complex trait defined by multiple components as plant population density, the number of pods per plant, the number of seeds per pods or the seed weight (Diepenbrock 2000). The potential seed yield of winter oilseed rape is determined since the end of the fall but depends on many factors and stresses occurring all along the crop life cycle as abiotic stresses (temperature, water and radiation), biotic stresses (pest, pathogens and weeds) or nutritional stresses, particularly with nitrogen (Rathke et al. 2006). All these constraints on winter oilseed rape seed yield result in an important environmental effect and *G* × *E* interaction that explained around 10% of the seed yield variation under French conditions (Bouchet et al. 2016; Corlouer et al. 2019). Numerous studies reported the genetic determinant of SY in rapeseed (reviewed by Delourme et al. 2018) and reported a high number of QTL (Shi et al. 2009; Raboanatahiry et al. 2018), thus confirming SY as highly polygenic. The comparison between QTL detected in different environments revealed some QTL being characterized as “stable” (i.e., detected across all environments) and QTL being characterized as “interactive” (i.e., specific to an environmental condition, Bouchet et al. 2016). However, the QTL specificity observed in a MET is rarely associated with the identification of the environmental features causing the observed adaptation.

In this context, this study aims at identifying the genetic determinants of seed yield stability in winter oilseed rape and at interpreting them in the light of environmental characteristics. We based our strategy on the analysis of winter oilseed rape accessions experimented across a multi-environment trial (MET-47) consisting in 47 environments representing the diversity of French growing conditions. First, we developed a comprehensive characterization of the MET-47 to identify the limiting factors that occurred. Then, to identify the genetic determinant of SY stability, a panel of 173 accessions was experimented in a sub-MET of 22 environments out of the 47 (MET-22). Envirotypes were defined among the MET-22 and corresponded to the clustering of the 22 environments according to their limiting factors pattern. Then, GWAS analyses were carried out using BLUE (Best Linear Unbiased Estimator) obtained for each genotype and each envirotype to identify QTL specific to environmental patterns. Finally, a genetic diversity analysis was carried out to decipher the potential impact of breeding for seed quality on the reduction of genetic diversity at those detected QTL that may limit adaptation to specific environmental conditions.

## Materials and methods

### Field network description

Field experiments were run in a multi-environment trial (MET-47) consisting of 47 environments defined as combinations of ‘year × location × nitrogen (N) fertilization’ in France between 2011 and 2018 (Supplemental Data 1). Each individual trial was conducted using classical crop management for winter oilseed rape (WOSR) with comprehensive protection against weeds, pests and pathogens. Optimal N fertilization was estimated using the balance sheet method (Rémy and Hébert 1977; Parnaudeau et al. 2009) for a target yield of 35 q ha^−1^ and applied in a subset of 19 environments defined as high N (N^+^). In contrast, a low N fertilization regime was applied in the remaining 28 environments defined as low N (N^−^) that corresponded to the N^+^ regime lowered by 80–100 kg ha^−1^ of N (Supplemental Data 1). Each environment was designed as a randomized complete block design with two to four replicates depending on the environment, with an individual plot area ranging from 6.75 to 14 m^2^. Mature dry seeds were harvested when the vegetative parts were fully senescent and the seeds were dark and hard. The targeted traits were the seed yield (SY in q ha^−1^) determined for each genotype in each trial and adjusted to 0% water content and 0% impurities, as well as the seed number (SN in seeds m^−2^) calculated according to the SY and the thousand seed weight (SN = SY × 100 000/TSW).

### Plant material

The WOSR genotypes “Aviso" and “Montego" were trialed over the whole MET-47 and therefore considered as probe genotypes for environmental characterization as reported by Corlouer et al. (2019). A diversity panel of 173 WOSR accessions (hereafter referred to as P173) was scored for SY and SN over a subset of 22 environments (11 N^+^ and 11 N^−^, called hereafter MET-22) run over the 2013–2014 and 2014–2015 growing seasons. The P173 accessions originated primarily from Western Europe and mostly represented commercial varieties released from 1959 to 2010 (Supplemental Data 2) with 31 accessions of the ‘++’ type (high contents in both erucic acid and glucosinolates; high C22:1, high GSL), 15 accessions of the ‘0+’ type (low C22:1, high GSL), 1 accession of the ‘+0’ type (high C22:1, low GSL) and 126 accessions of the ‘00’ type (low C22:1, low GSL) of which 46 were elite lines. Seeds were provided by the BrACySol Biological Resource Center or private seed companies. All accessions of the P173 population were produced in the same nursery at Le Rheu, France in 2010–2011 growth season to avoid seed lot biases. The P173 population was genotyped using the Brassica 60K Illumina Infinium™ array (Clarke et al. 2016) and an exome sequence capture assay (Leveugle et al. 2015). A total of 217,805 SNP were scored and validated for the current study using a threshold of 2.5% for the minor allele frequency (MAF) and of 10% for the missing values. The missing genotyping data were inferred using Beagle v3 software (Browning and Browning 2009). All the SNPs were physically anchored onto the latest *Brassica napus* reference genome of Darmor-*bzh* (Rousseau-Gueutin et al. 2020).

### Environmental characterization and envirotyping

Pedoclimatic indicators were calculated as described by Corlouer et al. (2019) and estimated for each of the key periods of the winter oilseed rape growing cycle. Briefly, these periods were fixed on meteorological data and the phenology of the probe genotypes. They covered fall (F), climatic winter (CW), bolting (B), seed number fixation (P300), reserve allocation to the pod (P600) and seed growth (P1000) stages. The 70 indicators correspond to four main categories: water stress, temperature, radiation and vernalization conditions (Corlouer et al. 2019). In addition, new indicators were developed to consider the contrasting N nutrition status (Supplemental Data 3). These included indicators related to the plant N status such as the nitrogen nutrition index (NNI; Colnenne et al. 1998) as well as the aerial dry biomass (ADB) and the aerial nitrogen quantity (AN), all scored at bolting stage (BBCH50, Lancashire et al. 1991) with a minimum delay of 2 weeks after the latest N supply. These values were measured on plants collected in the field on a surface of 1 m^2^ and are expressed in quantity per hectare. On the other hand, indicators related to the N fertilization management were also considered such as the total amount of N supplied to the crop (N_total_, in kg ha^−1^), and the water regime at the time of N supply was assessed considering the maximum number of days without rainfall during the 10 days preceding the N supply (DBI, dryness before input), the number of days without rainfall over 10 days after the N supply (DAI, dryness after input), the sum of the rainfalls over 10 days after the N supply (RF, rainfall), the number of days of leaching over 10 days after the N supply (RO, run off) and the mean value of the water soil content at the time of the N supply (from 3 days before up to 10 days after the input) expressed in percentage of the maximal water soil content (WSC_I). Given that N fertilization was provided in one, two or three applications depending on the local pedoclimatic conditions, we defined a unique indicator for each trial that considers the number of N supplies, calculated as following:

1 $${\text{Final}}\;{\text{indicator}} = \mathop \sum \limits_{i = 0}^{n} {\text{Indicator}}_{i} \times \frac{{N_{i} }}{{N_{{{\text{total}}}} }}$$

where Indicator_*i*_ is the given indicator (DBI, DAI, RF, RO or WSC_I) calculated at the *N* supply *i*, *N*_*i*_ is the amount of N brought to the environment at the application *i* and *N*_total_ the total *N* amount considering all the *N* applications. When no *N* fertilization was applied, the final indicators were fixed to 0. Missing indicators were imputed using the missMDA package (Josse and Husson 2016).

A total of 79 indicators (Supplemental Data 3) were considered in the present study. For a more precise analysis of the SY stability, an envirotyping was carried out according to the methodology proposed in Corlouer et al. (2019). Briefly, a univariate Partial Least Square (PLS) regression analysis was run at the MET-47 scale by regressing the mean seed yield of Aviso and Montego for each of the 47 environments on the 79 environmental indicators. A variable selection was performed to keep the reduced set of indicators that best explained seed yield variation, hereafter referred as limiting factors. Then, a hierarchical clustering was performed on the environments of the MET-22 to define envirotypes according to their pattern of limiting factors.

### Phenotypic data analyses

Trait analysis was run using three different mixed linear models that were fitted using the “lme4" (Bates et al. 2015) and ‘lmerTest’ packages (Kuznetsova et al. 2017), and corresponded to the three scales of analysis used in this study, i.e., the whole MET-22 scale, the envirotype scale (cluster of different environments presenting the same pattern of limiting factors) or the single environment scale. In the different models, the random effects are underlined in the equation, the others effects are considered as fixed.

A first linear mixed model (2) was fitted at the MET-22 scale including an effect of the envirotype to evaluate the impact of the envirotyping on the variance repartition.

2 $$Y_{ijkl} = \mu + G_{i} + En_{l} + En_{l\left( k \right)} + G_{i} \times En_{l} + G_{i} \times En_{l\left( k \right)} + \underline{{R_{{j\left( {l \times k} \right)}} }} + \underline{{\varepsilon_{ijkl} }}$$

where $Y_{ijkl}$ is the phenotypic value, $\mu$ is the population mean, $G_{i}$ stands for the effect of genotype i, $En_{l}$ for the envirotype l, $En_{l\left( k \right)}$ for the environment k nested in the envirotype l, $R_{{j\left( {l \times k} \right)}}$ for the replicate j, nested in the environment k and envirotype l; and $\varepsilon_{ijkl}$ is the residual.

Then, at the single environment scale, models (3) and (4) were used, and at the envirotype and at the MET-22 scale, models (5) and (6) were used to study the genotypic effect, to calculate the corresponding best linear unbiased estimators (BLUE), and to estimate trait heritability. These models are presented below.

Model (3) was fitted at the scale of each environment:

3 $$Y_{ij} = \mu + G_{i} + \underline{{R_{j} }} + \underline{{\varepsilon_{ij} }}$$

where $Y_{ij}$ is the phenotypic value, $\mu$ is the population mean, $G_{i}$ stands for the effect of genotype *i*, $R_{j}$ for the replicate *j* and $\varepsilon_{ij}$ is the residual. The replicate effect and the residual were declared as random. The heritability was calculated as:

4 $$h^{2} = \frac{{\sigma_{G}^{2} }}{{\sigma_{G}^{2} + \frac{{\sigma_{\varepsilon }^{2} }}{r}}}$$

where $\sigma_{G}^{2}$ is the genetic variance, $\sigma_{\varepsilon }^{2}$ the residual variance and *r* the number of replicates per genotype.

Model (5) was applied at the envirotype or at the MET-22 scale:

5 $$Y_{ijk} = \mu + G_{i} + E_{k} + G_{i} \times E_{k} + \underline{{R_{j\left( k \right)} }} + \underline{{\varepsilon_{ijk} }}$$

where $Y_{ijk}$ is the phenotypic value, $\mu$ is the population mean, $G_{i}$ stands for the effect of genotype *i*, $E_{k}$ for the environment *k*, $R_{j\left( k \right)}$ for the replicate *j*, nested in the environment *k* and $\varepsilon_{ijk}$ is the residual. The corresponding heritability was defined as follow with all terms declared as random in Eq. (5):

6 $$h^{2} = \frac{{\sigma_{G}^{2} }}{{\sigma_{G}^{2} + \frac{{\sigma_{G \times E}^{2} }}{t} + \frac{{\sigma_{\varepsilon }^{2} }}{r*e}}}$$

where $\sigma_{G}^{2}$ is the genetic variance,$\sigma_{G \times E}^{2}$ the *G* × *E* interaction variance, $\sigma_{\varepsilon }^{2}$ the residual variance, *e* the number of environment and *r* the number of replicates per genotype.

### GWAS analyses

The BLUE defined at each scale (environment, envirotype, MET-22) using models (3) and (5) for SY and SN was used to perform the genetic analyses. GWAS analyses were conducted using the FastLMM algorithm (Lippert et al. 2011) using a kinship matrix calculated for each linkage group as described by Rincent et al. (2014) and following the Astle algorithm (Astle and Balding 2009). A Bonferroni detection threshold was set to 10%, based on a corrected SNP population as proposed by the simpleM method developed by Gao et al. (2008). This method was based on the composite linkage disequilibrium (CLD) correlation between SNP. The CLD was used to calculate the effective number of independent tests (*M*_eff_). In this study, the *M*_eff_ was calculated and fixed to 19,886 SNP. We defined the threshold of highly significant associated SNP as:

7 $$t = - \log_{10} \left( {\frac{{\alpha_{G} }}{{M_{eff} }}} \right)$$

where *t* is the value of the value of the threshold, $\alpha_{G}$ is the threshold of the *p*-value and was fixed to 0.1 and *M*_eff_ was the effective population of SNP. The final threshold used was *t* = 5.30.

### Genetic diversity analysis

A genetic diversity was conducted at the associated loci detected through GWAS. This evaluated the potential impact of a major breeding event following the selection of varieties without glucosinolates in seeds. Therefore, two populations were confronted: a first population (GSL+) composed by genotypes with high contents of glucosinolate in seeds (> 18 µmol g^−1^) (46 WOSR, “++” and “0+”) and a second population (GSL-) composed by the remaining accessions of the P173 that present low glucosinolate content (< 18 µmol g^−1^) (127 WOSR, “+0” and “00”) (Supplemental Data 2). The nucleotide divergence statistic (π) and the mean Fst (Weir and Cockerham 1984) for the whole P173 and for the GSL+ population and the GSL– population were calculated using VCFtools v0.1.13 software (Danecek et al. 2011) for a window of 10 kb around each genomic region of interest.

## Results

### N stress was not the only factor impacting seed yield

**Fig. 1:**
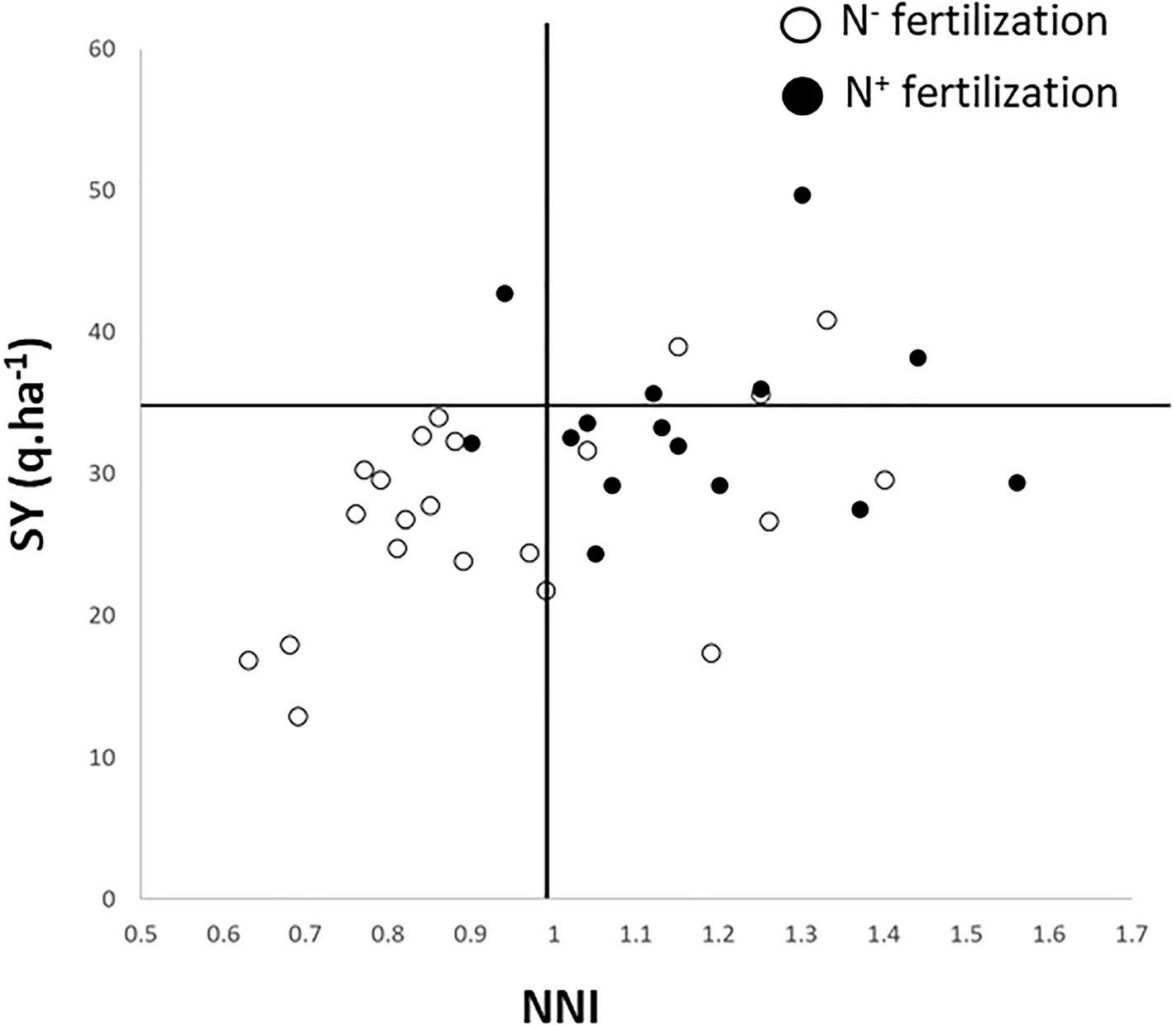
Relationship between mean values of seed yield (SY, *Y*-axis) and nitrogen nutrition index (NNI, *X*-axis) observed for the probe genotypes, Aviso and Montego, across the MET-47. Empty and plain circles correspond, respectively, to the N^−^ and N^+^ nitrogen fertilization regimes. A vertical line was added and corresponds to a NNI value of 1, above which environment are considered as not impacted by N stress. The horizontal bar corresponds to a SY of 35 q ha^−1^ which is the targeted yield for N^+^ conditions

A first overlook of the MET-47 was carried out by analyzing the relationship between the mean values of seed yield and NNI for Aviso and Montego per environment (Fig. 1). When focusing on environments defined as N^−^, it is to be noticed than NNI values ranged from 0.63 to 1.26, with NNI values exceeding 1.0 for 7 N^−^ -environments. Moreover, in two N^−^ environments (Lou18_N^−^ and Chr18_N^−^), the observed SY was even higher that the targeted value for N^+^ conditions. Thus, these results demonstrated the difficulty of implementing a nitrogen stress under field conditions. When considering the N^+^ environments, it can be observed that the targeted seed yield of 35 q ha^−1^ was achieved in 13 environments out of 19. However, in these 13 environments, NNI was always higher than 1 (except for LR13_N^+^), indicating the presence of other environmental factors that did impact seed yield. These results illustrated the need to consider a comprehensive environmental description of the trials to detect the factors that indeed affected SY.

### Identification of 13 environmental indicators limiting seed yield

MET-47 offered a unique chance to get insights into the SY most limiting factors. For that purpose, the set of 79 indicators was used to run a PLS regression analysis on the mean SY variation of Aviso and Montego for each of the 47 environments. SY-limiting factors corresponded to a set of 13 indicators (see Table 1 for a precise description). Predicted seed yields using the PLS regression model were strongly correlated to the observed seed yields (*R*^2^ = 0.87, RMSE = 2.6), thus validating the set of indicators. These 13 indicators covered the whole crop growing cycle from the climatic winter (CW) to the reserve allocation to the pod period (P600). Six indicators referred to the temperature (TMN_CW, TMAX_B, TMAX_FLO, HT_FLO, TMAX_P300, and TMIN_P600), two to the water status (WSC_MAX and WD_P600), one to the radiation (SSR_FLO), one to vernalization conditions (VERN) and three to nitrogen status (NNI, *N*_total_ and DAI; Table 2). Finally, the study of the Pearson correlations (Table 2, Supplemental Fig. 1) showed important correlations between the 13 identified indicators and indicators related to the reproductive periods of winter oilseed rape (FLO to P1000) and to the climatic winter period (Table 1), highlighting the key role of these periods on the elaboration of the final seed yield.

**Table 1:** Seed yield limiting indicators across the MET-47

Indicators	Description	Correlated indicators*
TMN_CW	Mean temperature during CW	–
TMAX_B	Maximal temperature registered during B	–
TMAX_FLO	Maximal temperature registered during FLO	SSR_CW (0.67); LT_B (-0.69); LT_FLO (-0.66)
HT_FLO	Number of days with high temperature (> 25 °C) at FLO	TMIN_CW (-0.7); TMN_FLO (0.66); LGDD_FLO (0.66); TMN_P600 (0.7)
TMAX_P300	Maximal temperature registered during P300	LGDD_CW (0.68); SSR_CW (0.67); LSR_CW (0.7); LT_FLO (-0.68); HT_P300 (0.68)
TMIN_P600	Minimal temperature registered during P600	TMIN_FLO (0.73); TMN_FLO (0.7); LT_FLO (-0.67); LGDD_FLO (0.7); TMN_P600 (0.78)
WD_P600	Number of days with WSC = 0 during P600	HT_F (0.66); WS_FLO (0.7); WSC_P300 (-0.68); WSC_P600 (-0.66)
WSC_MAX	Maximal water soil capacity	TMN_F (-0.67)
SSR_FLO	Sum of solar radiation during FLO	LSR_FLO (-0.67); TMAX_P1000 (0.69); HT_P1000 (0.65)
VERN	Optimal vernalization treatment	LT_CW (0.65)
NNI	Nitrogen nutrition index	AN (0.65); *N*_total_ (0.66)
N_*total*_	Nitrogen input	NNI (0.66)
DAI	Number of days of dryness after N supply	–

*Value of the Pearson correlation coefficient was indicated between brackets

**Table 2:** Results of the mixed linear model applied on the MET-22 to evaluate the impact of the envirotyping on variance repartition

Trait	Models	% Variance				
	Models (5)	*G*	*E*	*G* × *E*	*R*	*ε*
SY		24.7	55.5	5.8	3.8	10.1

	Model (2)	*G*	*En*	*En* × *E*	*G* × *En*	*G* × *En* × *E*	*R*	*ε*
SY		20	44.4	19.2	1.2	3.9	3.1	8.2

Data had been analyzed using linear model (2): $Y_{ijkl} = \mu + G_{i} + En_{l} + En_{l\left( k \right)} + G_{i} \times En_{l} + G_{i} \times En_{l\left( k \right)} + \underline{{R_{{j\left( {l \times k} \right)}} }} + \underline{{\varepsilon_{ijkl} }}$; with the genotype (*G*), the envirotype (*En*), the environment nested in the envirotype (*En*_*l*(*k*)_ or *En* × *E*), the genotype by envirotype interaction (*G* × *En*), the genotype by environment by envirotype interaction (*G*_*i*_ × *En*_*l*(*k*))_ or *G* × *En* × *E*) and the block effect nested in the environment, nested in the envirotype (R) and compared with the results of linear model (5): $Y_{ijk} = \mu + G_{i} + E_{k} + G_{i} \times E_{k} + \underline{{R_{j\left( k \right)} }} + \underline{{\varepsilon_{ijk} }}$, with the genotype (*G*), the environment (*E*), the genotype by environment interaction (*G* × *E*) and the block (*R*) nested in the environment

### MET-22 included four contrasting envirotypes

**Fig. 2:**
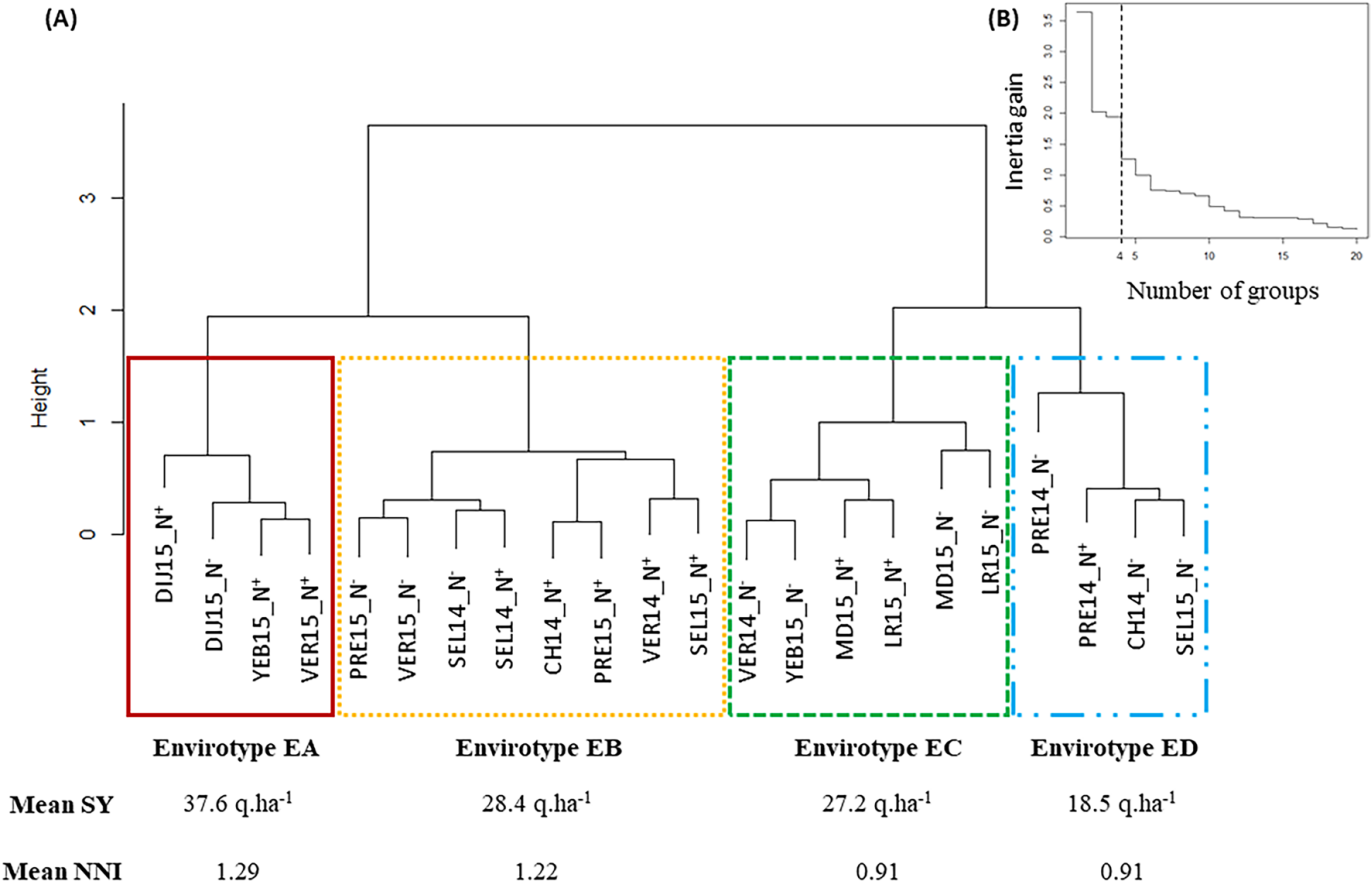
Envirotyping of the MET-22. **a** Dendrogram tree of the 22 environments of the MET-22 based on the PLS regression results. Mean seed yield (SY) and mean nitrogen nutrition index (NNI) values are indicated for each envirotype. **b** Variation of the inertia gain depending on the number of groups chosen for the hierarchical clustering. The dashed line represents the choice of groups number

**Fig. 3:**
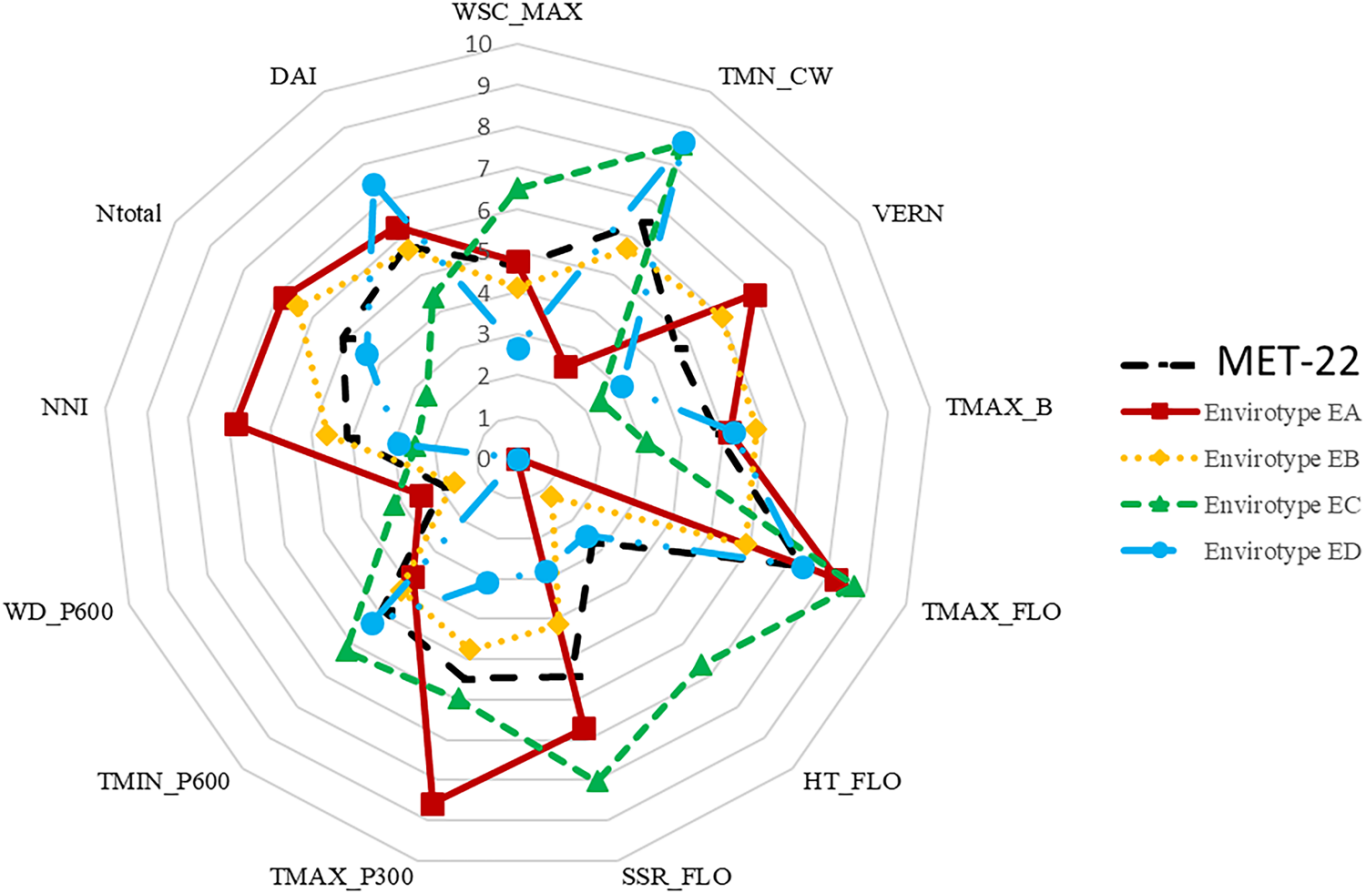
Envirotype description based on the seed yield limiting factors. The profile of each limiting factor is represented on a 0–10 scale for each envirotype as well as for the MET-22. Abbreviations of the indicators are as following: *TMN_CW* mean temperature at climatic winter, *VERN* vernalization condition, *TMAX_B* maximal temperature at bolting, *HT_FLO* number of days with high temperature (> 25 °C) at flowering, *SSR_FLO* sum of solar radiation at flowering, *TMAX_P300* maximal temperature during the seed number fixation period, *TMIN_P600* minimal temperature during the pod growth, *WD_P600* water deficit during the pod growth, *NNI* nitrogen nutrition index, *N*_total_ amount of nitrogen bring to the environment, *DAI* number of dry days after the N^−^ input, *WSC_MAX* maximal water soil capacity

The MET-22 is a subset of the MET-47 where 173 genotypes were trialed. The mean seed yield of the MET-22 was 30.35 q ha^−1^ (29.40 q ha^−1^ for the MET-47), and the mean NNI across the MET-22 was 1.06 (1.02 for the MET-47). The MET-22 description using the limiting indicators previously identified presented a similar profile to the MET-47 (Supplemental Fig. 2). A hierarchical clustering was performed on the environments of the MET-22 to define envirotypes according to their pattern of limiting indicators. Four envirotypes were defined (EA, EB, EC and ED; Fig. 2, Supplemental Data 1). Envirotype EA is composed of three N^+^ environments and one N^−^ environment. The mean seed yield observed across EA was 37.6 q ha^−1^, and the mean NNI was 1.29. This envirotype can therefore be defined as non N stressed and high yielding. The analysis of the limiting indicators pattern showed that EA, when compared to the mean MET-22, is characterized by lower temperature during winter, lower heat stress and solar radiation at flowering (HT_FLO), a higher temperature during the seed number fixation period (TMAX_P300) and a higher NNI (Fig. 3).

In contrast, envirotype ED was characterized by the lowest mean seed yield (18.5 q ha^−1^) and a mean NNI of 0.91 suggesting that the environments of this envirotype have been more stressed. ED gathered three N^−^ environments and one N^+^ environment. The limiting indicators pattern of envirotype ED is characterized by poor vernalization conditions (VERN), higher temperature during winter (TMN_CW), lower SSR_FLO and TMAX_P300 than the mean MET-22 (Fig. 3).

Finally, the two remaining envirotypes, EB and EC, presented mean seed yields of 28.4 and 27.2 q ha^−1^, respectively, while the mean MET-22 seed yield was 30.34 q ha^−1^. Envirotype EB was composed of eight environments (three N^−^ and five N^+^) and envirotype EC of six environments (four N^−^ and two N^+^). Envirotypes EC and EB distinguished from each other according to NNI (1.22 for EB and 0.92 for EC), N_total_, VRN, and conditions at flowering (HT_FLO and SSR_FLO). The profiles of limiting indicators showed that TMAX_FLO, HT_FLO, SSR_FLO, TMAX_P300, WSC_MAX and TMN_CW were lower in envirotype EB compared to the MET-22. Envirotype EC was characterized by higher HT_FLO, SSR_FLO, WSC_MAX and TMN_CW while DAI, NNI, N_total_, TMAX_B and VERN were lower than observed for the MET-22 (Fig. 3).

Our results demonstrated that the 22 environments of the MET-22 could be classified in four envirotypes: envirotype EA that can be considered as not stressed, envirotype ED considered as the most stressed, while envirotypes EB and EC showed moderate stress and mean SY similar to the mean SY observed at the MET-22 scale, but with a different pattern of limiting factors.

### Envirotyping explained up to 70% of environmental variation and 24.6% of *G* × *E*

To evaluate the impact of envirotyping on the variance distribution of the E and *G* × *E* effects, a linear model (2) was applied to SY. At the MET-22 scale, the envirotype effect (En) explained a main part of the environmental effect (70% for SY) as shown by the comparison of models (2) and (5) (Table 2). Genotype by envirotype interaction (*G* × *En*) explained 24.6% of the *G* × *E* interaction observed at the MET-22 scale. Within each envirotype, the *G* × *E* variation was reduced in envirotype EA (2.3% of total variation) when compared to the MET-22 (5.8%), but increased for envirotypes EB (8.7%) and particularly for ED (15.9%) (Table 3). As SY and SN were highly correlated (*r* = 0.94, *p*-value < 0.001), the envirotyping based on SY was used to study SN variation and its repartition. Similar results were observed and are reported on Table 4.

**Table 3:** Results of the mixed linear model applied for the MET-22 and for each envirotype considering all trials as confounded [linear model (5)] and heritabilities estimation

Trait	Group	Nb of environment^b^	% Variance^a^					*h*^2^
			*G*	*E*	*G* × *E*	*R*	*ε*	
SY	MET-22	22	24.7	55.5	5.8	3.8	10.1	0.98
	Envirotype EA	4	37.7	48.3	2.3	3.8	7.8	0.96
	Envirotype EB	8	38.5	34	8.7	4.8	14.1	0.95
	Envirotype EC	6	45.7	8.7	5.9	10.8	29	0.94
	Envirotype ED	4	28.9	34.8	15.9	5.3	15	0.83
SN	MET-22	18	33	40.4	8.1	4.9	13.5	0.98
	Envirotype EA	2	46.9	34.1	8.1	1.1	9.9	0.88
	Envirotype EB	7	49	27	9.4	2.1	12.5	0.96
	Envirotype EC	6	43.8	6.9	9.1	13.7	26.5	0.93
	Envirotype ED	3	29.6	32.5	11.5	7.4	19	0.81

Linear model (5): $Y_{ijk} = \mu + G_{i} + E_{k} + G_{i} \times E_{k} + \underline{{R_{j\left( k \right)} }} + \underline{{\varepsilon_{ijk} }}$, with the genotype (*G*), the environment (*E*), the genotype by environment interaction (*G* × *E*) and the block (*R*) nested in the trial. Heritabilities (*h*^2^) were estimated using Eq. (6):$h^{2} = \frac{{\sigma_{G}^{2} }}{{\sigma_{G}^{2} + \frac{{\sigma_{G \times E}^{2} }}{t} + \frac{{\sigma_{\varepsilon }^{2} }}{r*e}}}$

^a^Percentage of variance explained by each parameter

^b^Number of trials considered for the evaluation of the different effects and estimation of heritabilities

**Table 4:** Heritabilities estimated for each trait and each environment of the MET-22

Envirotype	Environment	*h*^2^	
		SY	SN
EA	Dij15_N^−^	0.90	–
	Dij15_N^+^	0.91	–
	Ver15_N^+^	0.91	–
	Yeb15_N^+^	0.92	0.93
EB	Ch14_N^+^	0.93	0.93
	Pre15_N^−^	0.81	0.85
	Pre15_N^+^	0.80	0.85
	Sel14_N^−^	0.94	0.93
	Sel14_N^+^	0.95	0.93
	Sel15_N^+^	0.63	–
	Ver14_N^+^	0.91	0.9
	Ver15_N^−^	0.86	–
EC	LR15_N^−^	0.85	0.88
	LR15_N^+^	0.87	0.9
	Md15_N^−^	0.67	0.53
	Md15_N^+^	0.74	0.65
	Ver14_N^−^	0.77	0.71
	Yeb15_N^−^	0.95	0.96
ED	Ch14_N^−^	0.81	0.78
	Pre14_N^−^	0.68	0.75
	Pre14_N^+^	0.87	0.87
	Sel15_N^−^	0.96	–

Components of the heritabilities were obtained using the linear model (3): $Y_{ij} = \mu + G_{i} + \underline{{R_{j} }} + \underline{{\varepsilon_{ij} }}$; Heritabilities (*h*^2^) were calculated according to Eq. (4): $h^{2} = \frac{{\sigma_{G}^{2} }}{{\sigma_{G}^{2} + \frac{{\sigma_{\varepsilon }^{2} }}{r}}}$

Heritabilities were calculated for SY and SN for each environment, for each envirotype and for the mean MET-22. Into more details, SY and SN were highly heritable (0.98 for the MET-22 for both traits) and heritabilities ranged from 0.83 to 0.96 for SY and from 0.81 to 0.86 for SN depending on the envirotype (Table 3). At the environment scale, heritabilities ranged from 0.63 to 0.96 for SY and from 0.53 to 0.86 for SN (Table 4). For most of the environments, SY and SN were highly heritable.

### Most of the SY-related QTL detected at the environment scale were specific of a single environment

Comparison of SY-QTL detected for each environment is a first step to characterize the genetic determinism of SY stability. Therefore, the BLUE obtained for each genotype and each environment using model (3) was used as input for GWAS analysis. A total of 87 SNP were detected showing a significant association with SY or SN considering all the analyses performed for each environment (Supplemental Data 4). These SNP were grouped into 11 QTL related to SY (QA03, QA05a, QA07a, QA07b, QA09, QC02, QC03a, QC04, QC08, QC09a and QC09b) and four QTL related to SN (QA05b, QA07a, QA09 and QA10) (Table 5). For almost all loci, the most frequent allele in the population was the favorable one except for QA03, QA07a and QC09b. Considering both traits, it is worth noting that all QTLs were specific to a single environment except QA07a, QA09, QA10, QC02, QC04 and QC09a. QA07a, QA10, QC02 and QC04 were only detected in two environments. The QA09 and QCA09a were detected in a large range of environments with six environments for QA09 and seven for QC09a, respectively.

**Table 5:** Description of the QTL detected for seed yield and seed number stability/instability

Name	Chromosome	Positions	Traits	Environments	Envirotypes	MET-22
QA03	A03	6,452,989	SY	Pre15_N^+(EB)^	–	–
QA05a	A05	2,078,385–2,080,519	SY	Chr14_N^+(EB)^	–	–
QA05b	A05	3,630,228	SN	Pre14_N^+(ED)^	–	–
QA07a	A07	138,830 – 1,009,271	SN	LR15_N^+(EC)^/Pre14_ N^−(ED)^	ED	–
		138,830 – 1,009,271	SY	LR15_N^+(EC)^		
QA07b	A07	20,033,023 – 20,033,025	SY	LR15_N^+(EC)^	–	–
QA09	A09	3,933,991 – 4,504,817	SY/SN	Yeb15_N^+(EA)^/Ver14_N^+(EB)^ Ch14_N^−(ED)^/Ch14_N^+(EB)^ Dij15_N^−(EA)^/LR15_N^+(EC)^	EA/ED	Yes
QA10	A10	2,515,307	SN	Pre14_N^−(ED)^/Ver14_N^−(EC)^	–	–
QC02	C02	60,063,320 – 60,615,209	SY	Ver14_N^+(EB)^/Yeb15_N^+(EA)^	–	–
QC03a	C03	20,140,168 – 20,809,663	SY	Sel15_N^+(EB)^	–	–
QC03b	C03	23,645,808	SN	-	EC	–
QC04	C04	6,033,108 – 6,289,953	SY	Dij15_N^+(EA)^/Pre15_N^−(EB)^	–	–
QC08	C08	37,326,511 – 37,326,814	SY	Ver14_N^+(EB)^	–	–
QC09a	C09	2,184,690 – 4,511,229	SN	–	EA/EB	–
QC09a	C09	2,184,690 – 4,511,229	SY	Ver14_N^+(EB)^/Sel14_N^−(EB)^ Md15_N^+(EC)^/Dij15_N^−(EA)^	EA/EB/EC	
				Yeb15_N^+(EA)^/Ch14_N^+(EB)^		
				LR15_N^+(EC)^		
QC09b	C09	51,377,310	SY	Ver14_N^−(EC)^	–	–

A QTL is defined as a region that can gather different individual QTLs detected at different scales (MET, envirotype, environment; Supplemental Data 4) with overlapping positions. The range of the individual positions observed for a dedicated QTL is indicated in the “Positions” column. The positions refer to the reference genome Darmor-*bzh* v10 version (Rousseau-Gueutin et al 2020). The “Traits” column refers to Seed Yield (SY) or Seed Number (SN), the environments, envirotypes and MET-22 indicated the different scales for which the considered QTL was detected

### Envirotyping highlighted five QTLs

BLUE calculated for each genotype and for each of the four envirotypes was used for GWAS analysis. In this way, the detected QTL will be linked to the envirotype characterization ran upon through their profile of limiting indicators.

QTL QC09a (Table 5) was detected for SY and SN and for three envirotypes (EA, EB and EC). It was also detected for SY in seven environments as previously mentioned (Table 5, Supplemental Data 4). It explained between 9.5 and 16.7% of SY variation depending on environment or envirotype; and 20.4% of SN variation for envirotype EA and 18.8% of SN variation for envirotype EB. The major allele was favorable to increase both traits (Supplemental Data 4).

The SN QTL QC03b (Table 5) was specifically detected in envirotype EC. It was not detected at the environmental scale. This QTL explained 21% of the SN variation (*R*^2^ = 0.21) in envirotype EC, and the major allele was the favorable one. However, confidence in this QTL is low as it is composed of a single SNP (Supplemental Data 4).

QA07a was detected for SN in envirotype ED on chromosome A07 (Table 5). This QTL was also detected in environment LR15_N^+^ (EC) and environment Pre14_N^−^ (ED) for SN and for SY in LR15_N^+^ (EC) (Table 5). This QTL explained 19.5% of the SN variation in envirotype ED, and the minor allele of the population was the favorable one. (Supplemental Data 4). QA07a could be considered as specific to stressed conditions.

Lastly, the QA09 and QC09a QTL, that were detected for a wide range of environments, were also detected for several envirotypes: EA and ED for QA09 and EA, EB and EC for QC09a.

### QA09 was the unique QTL detected across the MET-22

BLUE obtained for each genotype at the whole MET-22 scale was used to detect consistent QTL controlling SY and SN across environments. A set of 15 SNP was detected for SY on chromosome A09 (Supplemental Data 4) and consisted in the QTL QA09 (Table 5). QA09 explained 12% of the SY variation, and the favorable allele was the major allele. As previously shown, QA09 was also detected for SY at the envirotype scale for EA and ED (Table 5), and at the environment scale in six environments that belonged to EA (2), EB (2), EC (1) and ED (1) (Table 5, Supplemental Data 4). The same analysis carried out for SN revealed that locus QA09 was also detected, but only in envirotype EA. Thus, QA09 was qualified as a stable genomic region controlling SY and SY components across a wide range of environmental conditions.

### Two stable QTL (QA09 and QC09a) and an interactive QTL (QA07a) were revealed using a multi-scale QTL detection (environment, envirotype, MET)

Finally, the QTL analysis revealed two loci (QA09 and QC09a) that can be characterized as stable and a locus (QA07a) that can be characterized as interactive. QA09 was detected across the MET-22 in six environments and two envirotypes, and QC09a was detected in three envirotypes and seven individual environments. QA07a was specifically detected for the most stressed envirotype (ED) and could be a good candidate for breeding programs. The allelic diversity underlying this QTL must be further investigated to evaluate the genetic diversity available at this locus for breeding purposes. Indeed, the 46 elite lines of the panel already fixed the favorable allele for the two stable QTL QA09 and QC09a, but not for the interactive one QA07a (Supp Data 4). Ten other QTL were detected at the environment scale but could not be linked to a limiting factor profile.

To characterize the three main QTL detected, a mixed linear model was fitted to estimate the QTL × E interaction using the data obtained for the whole MET-22 and both for SY and SN. (Table 6). The QA09 × E interaction explained 31.9 and 34.5% of the total variance for SY and SN, respectively, whereas the genotype effect explained only 13.2 and 20.1% of the total variance for SY and SN, respectively. The QC09a × E interaction explained 31.9 and 38.5% of the total variance for SY and SN, respectively, whereas the genotype effect explained only 12.3 and 18.7% of the total variance for SY and SN, respectively. For the QA07a, that was qualified as interactive, the QTL × E effect explained less than 1% of variance for both SY and SN, this can be related to the overall small effect of this QTL at the MET-22 scale.

**Table 6:** Results of the mixed linear model applied at the MET-22 scale to test the effect of the QTL × E or the QTL × En interaction

	Seed yield (SY)			Seed number (SN)		
	QA09 (%)	QC09a (%)	QA07a (%)	QA09 (%)	QC09a (%)	QA07a (%)
Genotype	13.2	12.3	24.3	20.1	18.7	31.9
Trial	35.7	35.7	56	19.4	16.7	41.4
Block × Trial	4.1	4.2	3.7	5.4	5.3	4.9
QTL × Trial	31.9	31.9	0.3	34.5	38.5	0.9
residual	15.2	15.9	15.6	20.7	20.8	20.9

The three QTL QA09, QC09a and QA07a (described in Table 5) were considered. The following mixed Linear model was used $Y_{ijkl} = \mu + G_{i} + E_{k} + marker_{j} \times E_{k} + \underline{{R_{l\left( k \right)} }} + \underline{{\varepsilon_{ijkl} }}$, with the genotype (*G*), the environment (*E*), the QTL (marker), the genotype by environment interaction effect (*G* × *E*) and the block effect (R) nested in the trial., as proposed by Happ et al. (2021). The QTL effect is tested using the marker presenting the highest –log(*p*-value) within its confidence interval. In each case, the percentage of variance explained by the considered effect is indicated

### Diversity analysis at QA07a revealed a lack of favorable allele in the modern-grown varieties

The composition of the panel P173 (GSL+ vs GSL– lines) allowed highlighting the recent history of winter oilseed rape breeding, including rapid selection for low glucosinolate contents in seeds, and estimating its impact on genetic diversity at the whole genome scale, as well as at the scale of previously detected QTL. The F_ST_ analysis showed a slight differentiation between the GSL+ and GSL– populations (F_ST_ value of 0.078 at the whole genome scale, Table 7). However, the genetic differentiation was higher when considering specific chromosomes such as A09 and C09, already known to carry genes controlling seed glucosinolate pathway, but also for chromosomes A08, C02 and C03 (*F*_ST_ values > 0.1) (Table 7). No specific pattern was observed on the A07 chromosome harboring the interactive QA07a QTL for SN.

**Table 7:** Mean nucleotide diversity (*π*) and mean *F*_ST_ statistics per chromosome and per population

	Mean *π*			Mean *F*_ST_
	P173	GSL+	GSL–	GSL+ vs GSL–
Whole genome	1.69e^−04^	1.77e^−04^	1.61e^−04^	0.078
Sub-genome A	1.81e^−04^	1.92e^−04^	1.70e^−04^	0.070
Sub-genome C	1.58e^−04^	1.62e^−04^	1.51e^−04^	0.086
A01	1.34e^−04^	1.63e^−04^	1.19e^−04^	0.058
A02	1.55e^−04^	1.67e^−04^	1.47e^−04^	0.055
A03	2.03e^−04^	2.26e^−04^	1.89e^−04^	0.057
A04	1.65e^−04^	1.69e^−04^	1.60e^−04^	0.060
A05	1.99e^−04^	2.06e^−04^	1.88e^−04^	0.064
A06	2.17e^−04^	2.28e^−04^	2.06e^−04^	0.052
A07	2.03e^−04^	2.10e^−04^	1.98e^−04^	0.046
A08	1.96e^−04^	1.91e^−04^	1.81e^−04^	0.159
A09	1.45e^−04^	1.46e^−04^	1.30e^−04^	0.128
A10	1.76e^−04^	1.86e^−04^	1.69e^−04^	0.039
C01	1.73e^−04^	1.22e^−04^	1.85e^−04^	0.056
C02	1.31e^−04^	1.46e^−04^	1.21e^−04^	0.111
C03	1.85e^−04^	1.64e^−04^	1.85e^−04^	0.107
C04	1.43e^−04^	1.82e^−04^	1.23e^−04^	0.087
C05	1.40e^−04^	1.68e^−04^	1.33e^−04^	0.045
C06	1.60e^−04^	1.85e^−04^	1.42e^−04^	0.098
C07	1.75e^−04^	1.68e^−04^	1.70e^−04^	0.075
C08	1.59e^−04^	1.77e^−04^	1.54e^−04^	0.046
C09	1.29e^−04^	1.49e^−04^	1.16e^−04^	0.125

P173 corresponds to the whole diversity set (173 accessions). GSL+ corresponds to the 46 accessions of the P173 with high contents of glucosinolate in seeds (> 18 µmol g^−1^). GSL– corresponds to the 127 accessions of the P173 with low contents of glucosinolate in seeds (< 18 µmol g^−1^)

**Fig. 4:**
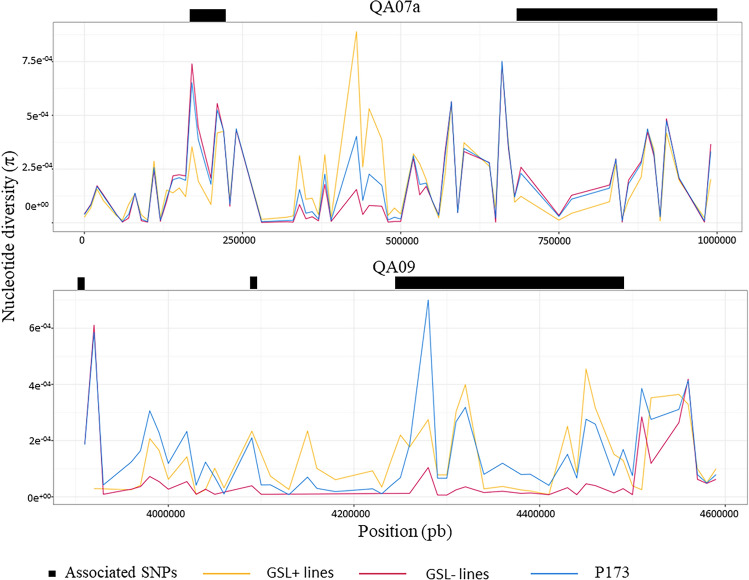
Nucleotide diversity (π) under QTL QA07a and QA09. The nucleotide diversity was calculated for all P173 population (blue curve), the GSL + (yellow curve) and GSL- (pink curve) accessions. The associated SNP for each QTL are indicated in black. The** π** index is calculated for windows of 10 Kbp (color figure online)

The nucleotide diversity index *π* was calculated at the whole genome scale and for each chromosome for both panel GSL+ and GSL–. Special attention was given to GSL- since this germplasm is more connected to the elite germplasm currently used by breeders. A scan of *π* values was also carried out targeting detected QTL regions. On average, lower nucleotide diversity was observed in the GSL + lines than in the GSL- lines excepted for chromosomes A07, A09, A10, C01, C07 and C08 (Table 7). However, the study of the *π* index at QTL QA07a showed a higher diversity in the GSL+ lines than in the GSL– (Fig. 4). These results showed a higher nucleotide diversity in GSL+ and consequently a lack of diversity in the GSL– germplasm, corresponding here to a deficit of the favorable allele.. In contrast, at the QTL QA09, a higher *π* value was observed in the GSL– illustrating a gain of nucleotide diversity induced by breeding at this locus (Fig. 4).

## Discussion

Based on a comprehensive environmental characterization of a MET composed of 22 locations, we identified a group of environments (ED) characterized by a combination of stresses (poor vernalization conditions, N stress and low temperature and radiation during flowering and grain filling period) that drastically impacted seed yield. In addition, we identified a QTL specific to these conditions (QA07a). This QTL was characterized by a reduced genetic diversity in the modern germplasm, when compared to older cultivars. Our analysis also highlighted two stable QTL controlling seed yields (QA09 and QC09a) that were expressed whatever the environmental conditions, as well as ten other QTL specifically detected in one or two single environments of the MET.

The question of crop adaptation and the underlying genetic determinism of crop stability is clearly a major challenge for agriculture for the coming decades. To answer this question, tools are being developed and the number of related studies raised drastically during the last years (van Eeuwijk et al. 2010, 2016). However, to our knowledge, only few studies dedicated to the analysis of *Brassica napus* genetic determinism of yield stability have been reported yet. The recent studies of yield stability specifically targeted the response of seed yield to water deficit (Zandberg et al. 2022; Raman et al. 2023) but did not address combination of different agro-pedoclimatic limiting factors. To address yield stability, authors usually test the QTL detected across different environmental conditions (Li et al. 2016; Lu et al. 2017; Zou et al. 2022). However, the proposed experimental designs are often limited to 4–6 environments, and *G* × *E* and stability genetic determinism are addressed by the confrontation of QTL detected for single environment to QTL detected across all the environments (Wang et al. 2018; Zheng et al. 2017; Lu et al. 2017; Li et al. 2020; Arifuzzaman et al. 2019; Pal et al. 2021; Sun et al. 2016; Gajardo et al. 2015) or by the meta-analysis of QTL detected for each environment using linkage analyses (Xie et al. 2020; Deng et al. 2019).

Within the MET-22, 25% of SY variation was due to a genetic effect, 55% to an environmental effect and 6% to *G* × *E*. This predominance of the E and G effects over the GxE effect supports the choice of grouping environments according to SY-limiting factors. This method made it possible to explain the E effect using envirotypes, and to examine the genetic determinant of SY in relation to environment characteristics. Indeed, the agro-pedoclimatic description of the MET-22 environments allowed explaining up to 70% of the environmental effect and only 24.6% of the *G* × *E* effect affecting seed yield. At the envirotype scale, the *G* × *E* part was reduced in the non-limiting envirotype EA (2.3%), but not in the most stressed envirotype ED (15.9%), when compared to MET-22 (5.8%). This result may be linked to the fact that the limiting factors used to group MET-22 were detected at the MET-47 scale. Indeed, although MET-22 and MET-47 present similar profiles in terms of limiting factors, slight differences were observed with regards to TMN-CW, N_total_ and DAI indicators. From an analytical point of view, this resulted in a loss of power in the decomposition of the *G* × *E* effect at the MET-22 scale. However, from an agronomic point of view, this resulted in a more representative list of limiting factors at the MET scale representing Western European rapeseed growing areas, leading to envirotypes more representative of growing conditions.

Overall, the envirotyping-based methodology allowed prioritizing three QTLs involved in the genetic determinism of seed yield stability across the 16 QTL detected. These three QTLs have also been reported by Bouchet et al. (2016) genetic germplasm related to the P173 population used here. The stable QTL QA09 was also reported in different studies for seed yield-related traits as summed up by Raboanatahiry et al. (2018), whereas no-colocalization was found in the literature for QC09a. The interactive QTL QA07a may correspond to the QTL detected previously for branching number (Zhao et al. 2016) or thousand seed weight and plant height (Quijada et al. 2006; Udall et al. 2006). The 13 remaining SY-related QTLs were only detected in one to two environments. This pattern of QTL specificity to a unique environment is widely reported in literature (Bouchet et al. 2016; Garin et al. 2020) and offers a first approach to QTL × E interactions, but the robustness of these specific QTL is also questionnable. The methodology that we proposed in this study was helpful to prioritize QTL and link the QTL with agro-pedoclimatic scenarii.

The low level of coincidence between our results and the QTL reported in the literature can result from differences in terms of genetic material used for GWAS or linkage studies. Indeed, most of studies reporting QTL for SY-related traits were carried out using spring type germplasm experimented in the field under short growth cycle conditions, unlike the winter oilseed rape germplasm used for this study. Winter and spring germplasms went through separated breeding history that can explain the differences of QTL detected, driven through selection for different breeding targets. Thus, broadening the genetic diversity considered for GWAS may help identifying additional seed yield-related QTL. For example, adding semi-winter accessions, more likely to withstand western Europe growing conditions, is a promising way to increase the population resolution by reducing the extent of linkage disequilibrium. A second way to improve the power of GWAS consists in increasing the number of genomic markers. However, recent genomics advances already led to a common use of resolutive genotyping resources such as the 60K Illumina array in most of the genetic studies (Clarke et al. 2016). Here, we developed and used a novel and dense genomic resource based on whole genome exome capture to characterize the population (Leveugle et al. 2015). This resource provided 217,805 SNP covering the entire genome and therefore increased the resolution of the QTL regions as a higher number SNP were detected per genomic region. Due to this genomic resource, the genome can be decomposed into 6763 haploblocks that presented a mean size of 48kb and that were in average composed of 28 SNPs (Supp Data 5). We also could precisely describe and capture the LD pattern of the P173 WOSR panel (Supp Fig. 3). This consists in an important resource, especially when dealing with complex traits such as seed yield.

To facilitate interpretation of QTL stability, approaches have already been proposed based on clustering of environments within a MET for other species. This involves grouping individual environments into mega-environments such that genotypes exhibit similar behaviors in a mega-environment and may differ between mega-environments.. Thus, Moreau et al. (2004) proposed a clustering of environments according to the *G* × *E* interaction matrix and were able to explain the specificity of QTL according to climatic or water stress conditions. More recently, the use of factor analytic models has been proposed and is specifically devoted to GxE decomposition among MET (Smith et al 2021). This method is highly resolutive in explaining GxE and in building cluster that minimize within GxE. The use of this methodology could have allowed to detect more “interactive” QTL; it is to say QTL presenting higher QTLxE effect. However, from an agronomic point of view, it is more difficult to link the cluster characteristics to environmental stresses experienced by plants. Millet et al. (2016) and Touzy et al. (2019) proposed to cluster the environments of a MET according to drought and/or heat scenarii and then to study the pattern of QTL effects from one scenario to another. Most of SY-related QTL presented significant interaction with the climatic scenario and for some of them, the favorable allele changed according to the scenario (Millet et al. 2016). Our study supports the interest of clustering the environments to identify and interpret QTL effects. In this study, we identified QTL (QA07a, QC03b) present in given envirotypes (ED and EC, respectively) and absent for others, a pattern also revealed in maize by Millet et al. (2016) and Touzy et al. (2019) when opposing contrasting scenarii. However, as opposed to Millet et al. (2016), we did not record any change of the favorable allele at a given QTL, depending on the envirotype. These two last studies focused their environments clustering on a priori defined stresses (water and temperature) and developed a targeted characterization of the environments of the MET. For winter oilseed rape, the 11-month growth cycle makes it more difficult to focus on a dedicated stress, and we therefore choose an alternative method that reports a posteriori the main combinations of environmental factors that did impact seed yield. Even if nitrogen input was managed to be one of the main limiting factors, it was clearly shown that it had to be considered in combination with others stress (radiation, temperature, …) occurring during the crop cycle, thus making ineffective an a priori clustering based on nitrogen indicators only. Moreover, Ravier et al. (2017) showed that N defiencies, even intense, do not always affect seed yield, especially if they occur early during the wheat growth cycle. This method was also successfully used to identify limiting factors occurring over a MET for barley (Beillouin et al. 2018) and highlights the need for indicators to account for potential stresses occurring in the field.

In this study, the envirotype ED corresponded to the combination of limiting factors that impacted the most seed yield. Its limiting factors targeted different phases of the crop cycle: winter (fulfillment of vernalization requirements and high temperatures during winter), bolting (N fertilization), flowering (lack of solar radiation) and grain filling (lower temperature). This high impacting combination of stress was observed for the four environments of envirotype ED. Individually, each of these factors has already been shown to impact seed yield: thermal stress during flowering affects flower fertility, pod number and seed number (Morrison 1993; Angadi et al. 2000; Young et al. 2004); a poor vernalization conditions could lead to delayed or no flowering (Ferreira et al. 1995; Chandler et al. 2005) and N limitation is also known to impact seed yield (Rathke et al. 2006). Here, we were able to identify that these different stresses co-occurred in the field and truly impact seed yield. Excepting N stress, the main limiting factors in ED corresponded to climatic factors.

We suggest that combining envirotyping and QTL analysis must be considered an effective approach, enabling the identification of both agro-pedoclimatic indicators and QTL involved in yield stability. QTL with the highest effects and qualified as "stable" may nevertheless present significant QTL × E interactions. However, they are detected in most environments and envirotypes, with the favorable allele being the same whatever the environment considered, which underlines their interest in improving yield for a large range of environmental conditions. Specific QTLs such as QA07a presented smaller effect and were not significantly involved in QTL × E at the whole MET-22 scale. However, this QTL presented a specific interest for a specific combination of limiting agro-pedoclimatic conditions that was observed in the envirotype ED. The analyses performed at the MET-22 scale or directly at the single environment scale were not consistent to highlight this genomic region. The molecular diversity as this locus indicated that the GSL cultivars did not fix the favorable allele, demonstrating the interest of the GSL+ cultivars as valuable source of genetic diversity for improving seed yield and its stability. This was confirmed by the fact that the elite lines already fixed the favorable allele for QTL QA09 and QC09 but not for QTL QA07a (supp Data 4). It is to be noticed that the QA07a does not colocalize with the QTL for glucosinolate content on the A07 chromosome (Tang et al 2023). This QTL indeed colocalizes with the QA07b that was detected for SY only in LR15^+^. Therefore, at the QA07a region, the favorable allele is more frequent in the “++” germplasm but the underlying genes might not be related to the glucosinolate pathway.

For further breeding programs, there is a particular interest in validating the four envirotypes described in the present study for a wider range of agro-pedoclimatic conditions. Indeed, a posteriori analysis of larger climatic datasets at the same locations, coupled with crop physiological models, such as AZODYN-colza (Jeuffroy et al. 2006), may conduct to an estimation of the frequency of these four specific envirotypes across growing seasons. Envirotype ED can consist in a new target environment for breeding if it occurs rather frequently within the rapeseed production area. This strategy could also help redesigning multi-environment trials for WOSR breeding, for instance by discarding redundant locations, reducing experimental costs and by maximizing the opportunities of desired envirotypes/pedoclimatic scenario within a MET. The envirotyping approach can also lead to an estimation of a similarity matrix of a MET locations, according to their limiting factors pattern. In silico experiments will be useful to test a wide range of genotypes and environmental conditions such as described by Wang et al. (2023) to validate our results. This can be a clue to identify accurate match between dedicated genotypes and environments (Resende et al. 2021) leading, notably, to better product placement for the seed industry.

### Supplementary Information

Below is the link to the electronic supplementary material.

Supplementary file1 (XLSX 1109 KB)

## Data Availability

The datasets generated and analyzed in this study are available using the following link (https://entrepot.recherche.data.gouv.fr/dataset.xhtml?persistentId=doi:10.57745/0TWHMA).
